# Screening of a Novel Solvent for Optimum Extraction of Anionic Surfactants in Water

**DOI:** 10.3390/toxics10020080

**Published:** 2022-02-08

**Authors:** Jung-Hwan Yoon, Yong Geon Shin, Hyuck Soo Kim, M. B. Kirkham, Jae E. Yang

**Affiliations:** 1 Kangwon Institute of Inclusive Technology, Kangwon National University, Chuncheon 24341, Korea; yoonfnfg@hanmail.net; 2 Department of Biological Environment, Kangwon National University, Chuncheon 24341, Korea; kimhs25@kangwon.ac.kr; 3 Gangwon Institute of Health and Environment, Chuncheon 24203, Korea; sindosa68@korea.kr; 4 Department of Agronomy, Kansas State University, Manhattan, KS 66506-0110, USA; kirkhammb@gmail.com

**Keywords:** anionic surfactants, chloroform, extraction, methyl isobutyl ketone, 1,2-dichloroethane

## Abstract

Anionic surfactants (AS) are detrimental aquatic pollutants due to their well-characterized toxicity to aquatic organisms. The concentration of AS in aquatic environments is increasing because of their extensive use in many industries and households. The standard reference method for AS analysis is to determine a methylene blue active substance (MBAS) complex formed between AS and the methylene blue (MB) cation by using chloroform. However, chloroform has a low AS extraction efficiency and other limiting properties, such as a high density and volatility, which make the conventional AS analytical method time-consuming and labor-intensive. In an effort to replace the use of chloroform, this study was carried out to screen novel solvents for their ability to extract AS in water samples. Criteria were based on AS extraction efficiency, physicochemical properties, and the stability of the solvent under different environmental conditions. Organic solvents, such as methyl isobutyl ketone (MIBK), 1,2-dichloroethane (DCE), dichloromethane, benzene, and n-hexane, were assessed. In extraction of the anionic surfactant sodium dodecyl sulfate (SDS), the mixture of MIBK-DCE (3:1) proved to be an optimum solvent as an alternative to chloroform. It not only enhanced SDS extractability but also improved properties, such as having a lower volatility, a lower density than water, and a quicker phase separation. Among solvents screened, no one single solvent in SDS extraction could meet such criteria. The performance of the MIBK-DCE (3:1) mixture in SDS extraction was stable, irrespective of pH and ionic strength of the SDS solution, washing process, and presence of cations. Anionic interference from halogen and polyatomic and organic anions in SDS extraction by MIBK-DCE (3:1) existed only at an elevated concentration, which is not occurring in the natural aquatic environment. Results demonstrated that a MIBK-DCE (3:1) mixture solvent could be used in AS analysis for a wide range of aquatic samples and it could be the basis for the development of a new analytical method to replace conventional chloroform.

## 1. Introduction

Surfactants are considered to be one of the most undesirable contaminants in aquatic environments because of their well-characterized adverse impacts on aquatic organisms as well as terrestrial ecosystems [1,2,3,4,5,6,7]. Elevated concentrations of surfactants in the environment are due to their extensive use in many industries and households and their discharge from wastewater [1,5,8].

Surfactants have amphiphilic characteristics and consist of a hydrophilic head and a hydrophobic tail component. They are categorized, depending on the charges of the hydrophilic head, into anionic, cationic, amphoteric, and nonionic forms [5,9,10]. The amphiphilic property of surfactants can reduce the interfacial tension between two liquid phases or a solid surface and liquid phase [9,11,12,13]. This renders surfactants as organic compounds that are the most highly produced and consumed, and they are widely used as detergents, solubilizers, wetting agents, cosmetics, and foaming agents [5]. 

Among surfactants, anionic surfactants (AS) are used more frequently in care products and household cleaning products than other types of surfactants, because of their low cost, high foaming efficiency, and, thus, high washing efficiency even at a low temperature [3,9,10,14]. Sodium dodecyl sulfate (SDS) and linear alkylbenzene sulfonates (LAS) are popular anionic detergents that are used worldwide [5,6,9,15]. Excessive use of AS has led to the discharge of contaminated wastewater into surface water, and the residual AS can further disperse in water, sediments, biota, and soil [5,8]. As a result, there is interest in determining AS residues in environmental samples [9,12,13,15,16,17,18,19,20]. 

Residual concentrations of surfactants in the environment are increasing [5,7]. Although a major proportion of surfactants can undergo aerobic or anaerobic degradation in natural aquatic environments and at wastewater treatment plants, concentrations of surfactants in wastewaters have been reported in the μg L^−1^~mg L^−1^ ranges that, in many cases, exceed the regulatory level [5,21,22,23,24,25,26,27]. The Korea Ministry of Environment [28] sets the regulatory standard of AS in surface water below 0.5 mg L^−1^ and the wastewater discharge allowance at 3 and 5 mg L^−1^ in clean-water-conservation areas and other areas, respectively. 

The official reference method for AS analysis in environmental samples relies on the use of chloroform and methylene blue [16,19,21,29,30,31,32,33,34,35]. It is a colorimetric method to analyze the methylene blue active substance (MBAS) that is formed between AS and methylene blue, and it is a blue-colored chloroform soluble ion-pair complex [3,18,19,29,30,31]. 

Use of an efficient organic solvent to recover AS in environmental samples is the prerequisite step for quantitative analysis. The official reference method using chloroform has the advantage of detecting low concentrations of AS in water [29,30]. However, chloroform shows a low AS extraction efficiency and has a relatively high density and volatility, and the resulting MBAS is instable at high temperatures and pHs [15,18,20,29,36,37]. The analytical procedure using chloroform requires repeated extractions by chloroform, is quite complicated and time-consuming, and requires a high volume of chloroform and much glassware [16,19,29,32,33,36]. Elevated concentrations of the Cl^−^ cause interference in the method, and, thus, it is limited in its application for analysis of AS in sea water [3,22,27,38]. Accordingly, to cope with such limitations that analytical procedures with chloroform have, an efficient solvent for AS determination in various water samples needs to be found.

The objective of this study was to screen novel organic solvents, which can be used to replace chloroform, for extracting anionic surfactants efficiently and rapidly in environmental water samples. The adequacy of the screened solvents was evaluated based on the AS extraction efficiency as well as its independence of effects of various environmental factors, such as pH, ionic strength, and interference from cations and anions.

## 2. Materials and Methods

### 2.1. Reagents

Sodium dodecyl sulfate (SDS: CH_3_[CH_2_]_11_OSO_3_Na, 99.0%) was purchased from Wako Pure Chemicals (Tokyo, Japan) and used as the standard anionic surfactant. Solvents to compare the AS extraction efficiency in this study were chloroform (CHCl_3_, 99.8%), methyl isobutyl ketone (MIBK: CH_3_COC_4_H_9_, 99.0%), 1,2-dichloroethane (DCE: ClC_2_H_4_Cl, 99.8%), dichloromethane (CH_2_Cl_2_, 99.9%), benzene (C_6_H_6_, 99.5%), and n-hexane (C_6_H_14_, 96.0%). Methylene blue (MB: C_16_H_18_N_3_SCl·3H_2_O, 97.0%) and the above solvents were purchased from Wako Pure Chemicals (Tokyo, Japan), except for DCE and MIBK, which were obtained from Junsei Chemical (Tokyo, Japan) and Daejung Chemical (Siheung, Korea), respectively. Concentration of MB as a cationic dye was prepared at 0.025% solution. All chemicals used were reagent grade.

### 2.2. Physiochemical Properties of Organic Solvents

Physicochemical properties of the selected organic solvents, including density, health hazard, vapor pressure, and odor threshold, were surveyed from references. This study measured the volatilization rate (mL min^−1^), in which each organic solvent (25 mL) was transferred to a previously dried and weighed aluminum dish and the dishes were weighed every 5 min for 2 h in an environment-controlled chamber. This measurement was triplicated.

### 2.3. SDS Extraction Efficiency by an Individual Solvent

The SDS standard solution was prepared at a concentration of 0.8 mg L^−1^ using distilled water, and 100 mL of this SDS solution was placed into 7 different separatory funnels. Then 5 mL of 0.025% methylene blue solution and 50 mL of each solvent, including chloroform, DCE, MIBK, dichloromethane, benzene, the n-hexane, and distilled water (control), were added to each separatory funnel and shaken for 3 min. Afterward, phase-separation time was recorded. After decanting the majority of the water layer, the solvent layers were filtered through water repellent phase separating paper (Whatman^®^ 1PS, Sigma-Aldrich, St. Louis, MO, USA). The maximum absorbance of the extracted methylene blue active substance (MBAS) and corresponding wavelength were measured with a UV/Vis spectrophotometer (DU800, Beckman Coulter, Indianapolis, IN, USA). The extraction efficiency of each organic solvent was determined based on the concentration recovery (%) at the maximum absorption wavelength. All measurements were triplicated.

### 2.4. SDS Extraction Efficiency of SDS by a Mixture of Organic Solvents

To screen novel solvents for SDS extraction, in an effort to replace the use of chloroform, the use of mixed solvents was investigated in addition to using a single solvent. With the use of a single solvent, it was impossible to meet the physicochemical properties of the extracting solvent that are required for optimum extraction of SDS in water samples. The optimum criteria for the screened solvent mixtures considered in this study were high SDS extraction efficiency, short phase-separation time between water and solvent layers, low volatility, and density lower than water. If density of a mixed solvent is lower than that of water, phase separation becomes much easier.

Two of six solvents tested in this study were mixed as a factorial combination and their efficiencies were determined through preliminary tests (all data not shown). The optimum combination was selected based on the above-stated criteria. The screened solvents satisfying the above criteria were MIBK and DCE. To find the optimum mixing ratio of the two solvents, 10 different mixing ratios (MIBK:DCE = 100:0, 95:5, 90:10, 85:15, 80:20, 75:25, 70:30, 65:35, 60:40) were tested. Then, the SDS extraction efficiency and the above-mentioned criteria were compared with those of chloroform. All measurements were triplicated.

### 2.5. Washing Effects on SDS Extraction by Screened Solvent

In the official reference method for determining AS, a washing process in the solvent layer after phase separation is required to remove impurities [29,30,36]. This study examined the necessity of the washing step on SDS extraction efficiency using the screened solvents, including DCE and MIBK. Chloroform was included for comparison. For this procedure, two separatory funnels for each solvent (‘A’ and ‘B’ batches) were prepared. Then the prepared SDS standard solutions were added, followed by the addition of 0.025% methylene blue solution (5 mL) and each solvent (50 mL). After 3 min., in ‘A’ batch, the layer of the extracting solvent was filtered through Whatman^®^ 1PS phase separator filter paper (Sigma-Aldrich, St. Louis, MO, USA) without washing, and, in ‘B’ batch, the layer of extraction solvent was similarly filtered after washing it once with distilled water. The maximum absorbance and the corresponding wavelength of the extracted MBAS by each solvent were measured and compared. All measurements were triplicated.

### 2.6. Factors Affecting SDS Extraction Efficiency by the Mixed Solvent

#### 2.6.1. pH Effects

Five mL of SDS solution (20 mg L^−1^) and 50 mL distilled water were transferred to a 100 mL volumetric flask. Solution pH was adjusted between 2~12 with dilute H_2_SO_4_ and NaOH, keeping the ionic strength at 0.32 M with 1 M Na_2_SO_4_. Final volume was adjusted to 100 mL with distilled water. After that, the 0.025% methylene blue solution (5 mL) and the selected MIBK-DCE mixture solvent (50 mL) were added to an aliquot of SDS standard solution and shaken for one min. After phase separation, the water layer was removed and the solvent layer was washed with distilled water and filtered through Whatman^®^ 1PS phase separator filter paper (Sigma-Aldrich, St. Louis, MO, USA). The absorbance of extracts was measured with a UV/Vis spectrophotometer (DU800, Beckman Coulter, Indianapolis, IN, USA) at 658 nm. All measurements were triplicated.

#### 2.6.2. Ionic Strength Effects

To determine the effect of ionic strength, the same experimental procedure for pH effects was followed after adjusting the ionic strength of the reacting solutions to 0, 0.01, 0.02, 0.05, 0.1, 0.2, 0.5, 1.0, and 2.0 M using dilute MgSO_4_ solution. All measurements were triplicated.

#### 2.6.3. Interference by Cations for SDS Extraction Efficiency 

To test interference by cations (Na^+^, K^+^, Mg_2_^+^, and NH_4_^+^) on SDS extractability by the mixed solvent, concentration of each of the respective cationic solutions was maintained at 0.1 M using Na_2_SO_4_, K_2_SO_4_, MgSO_4_, or (NH_4_)_2_SO_4_. The SDS standard solution and MIBK-DCE (3:1) solvent were mixed. The SDS extraction efficiency was determined using the same procedure as that for pH effects. Relative interference (%) by cations was assessed based on the recovery concentration of SDS in a cation-treated solution as compared to that of the control (100%). All measurements were triplicated.

#### 2.6.4. Interference by Anions for SDS Extraction Efficiency 

To test of interference by anions, solutions of NaF, NaCl, KBr, KI, NaNO_2_, KNO_3_, KH_2_PO_4_, KCN, NaHCO_3_, sodium acetate, trisodium citrate, sodium salicylate, potassium biphthalate, or potassium sodium tartrate were tested using the same procedure as that for pH effects. The degree of interference by anions was assessed by the interferential strength (IS: Equation (1)). This equation estimates the relative intensity of MBAS from the SDS and anion-treated solution (M) as compared to the intensity of MBAS from 1.0 mg L^−1^ SDS (3.5 × 10^−6^ M) which is set as 1. Thus, the higher the IS value is, the higher interference by anions.

(1) $$\mathrm{Interferential}\;\mathrm{strength}\;\left(\mathrm{IS}\right)=\frac{[\mathrm{MBAS}\left(\frac{\mathrm{mg}}{L}\right)\times [\frac{3.5\times 10^{-6}M\;\mathrm{SDS}}{\mathrm{SDS}\left(1\;\frac{\mathrm{mg}}{L}\right)}]]}{\left[\mathrm{concentration}\;\mathrm{of}\;\mathrm{interfering}\;\mathrm{ion}\;\left[M\right]\right]},$$

## 3. Results

### 3.1. SDS Extraction Efficiency by an Individual Solvent

An ideal solvent for determination of SDS in water samples should have such properties as a high extractability, a short phase-separation time, a low volatility, a low solubility in water, a lower density than water, and a lower potential health hazard [16,18,20,34,39]. However, chloroform falls short of satisfying such conditions [12,13,16,18,19], but it is still used widely as the main solvent for AS analysis. Feiterira et al. [34] and Yeerum et al. [35] used polyurethane foam (PUF), in an attempt to replace the use of chloroform. They measured the colored MBAS retained on a PUF surface directly using a digital image scanner. These methods require, however, adjusting pH and ionic strength and buffering at a certain pH. They also have a higher detection limit. 

This study screened six solvents (chloroform, dichloromethane, MIBK, n-hexane, benzene, and 1,2-dichloroethane) to select which solvent is better than chloroform for SDS extraction. Extraction efficiencies and properties were compared. Table 1 gives the absorbance of the standard SDS solution (0.8 mg L^−1^) and the phase-separation time when the SDS was extracted by six different solvents. 

After compensating for the absorbance of the blank, the absorbances of SDS solutions extracted by 1,2-dichloroethane (DCE), MIBK, and dichloromethane were higher than the absorbance of the chloroform extraction, indicating higher SDS extractability (Table 1). However, benzene and n-hexane had low or no absorbance compared to chloroform, and they were eliminated from further consideration. 

As shown in Table 1, the range of phase-separation time for chloroform was 12~22 min. Compared to chloroform, treatments of MIBK, n-hexane, and benzene had a shorter phase-separation time (1~3 min), whereas treatments with dichloromethane and 1,2-dichloroethane resulted in a longer separation time. After a complete separation, the maximum absorption wavelengths of SDS solutions from each solvent were similar and were in the range of 652~659 nm (Table 1). 

Based on these results, DCE (1,2-dichloroethane), MIBK, and dichloromethane were screened as candidates for further testing, even though DCE and dichloromethane had longer phase-separation times than chloroform. 

### 3.2. Physicochemical Properties of Solvents

Physicochemical properties of the solvents (Table 2) govern the analytical time, volume of solvents used, ease of handling of analyses, and experimental errors [16,18,19,34,39]. As described before, criteria for an optimum solvent for AS extraction need to include density and volatility.

If the density of the solvent is greater than water, phase separation yields an extractant layer on the bottom and an aqueous layer on the top between the two phases. Consequently, the analytical process can be complicated and time-consuming due to the fact that a different separatory funnel is required for the washing process, which results in a longer time for analysis [18,19,29,36]. In contrast, if the solvent has a lower density than that of water, the extraction and the washing process can be done using only one separatory funnel, thereby reducing the analytical time and labor. 

If the extractant has a high volatility, the vapor should be removed frequently when the extractant is mixed with the sample, because it can increase the risk of human exposure and result in a loss of MBAS together with the chloroform vapor. Furthermore, since the extract volume could be reduced by volatilization during the analysis, the solvent volume must be made constant before absorbance measurement [29,36]. 

As shown in Table 2, only MIBK had density (~0.8 g mL^−1^) lower than water. The vapor pressure of MIBK was the lowest at 20 °C, and the vapor pressure of DCE, chloroform, and dichloromethane were 4, 11, and 23 times higher than that of MIBK, respectively. The volatilization rates of MIBK, DCE, chloroform, and dichloromethane were 0.04, 0.12, 0.28, and 0.50 mL min^−1^, respectively. Judging from the boiling points, MIBK and DCE are heat stable. As far as safety is concerned, MIBK was considered as non-carcinogenic to human health, but other solvents were classified in the B2 class (Table 2). 

Results demonstrated that, when compared to chloroform, MIBK was the most suitable solvent for AS analysis, followed by DCE. Therefore, MIBK and DCE were selected for the next experiment. 

### 3.3. Effects of Washing Process on SDS Extraction

**Figure 1:**
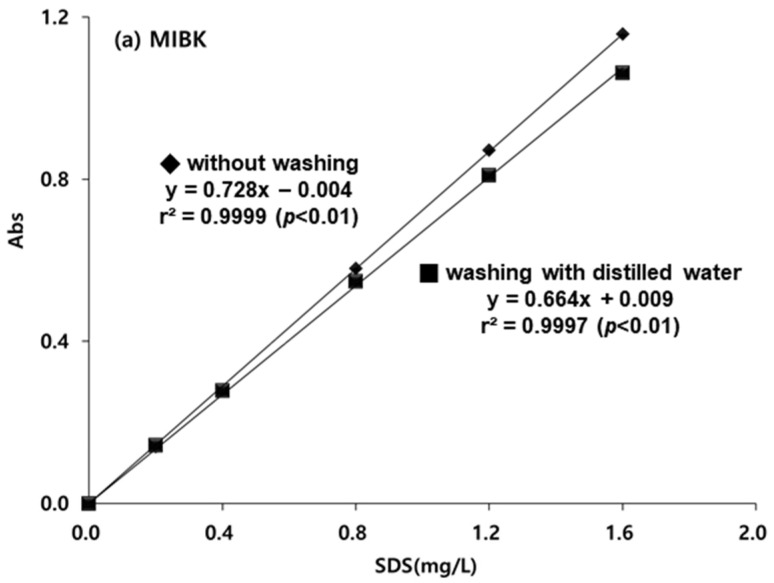

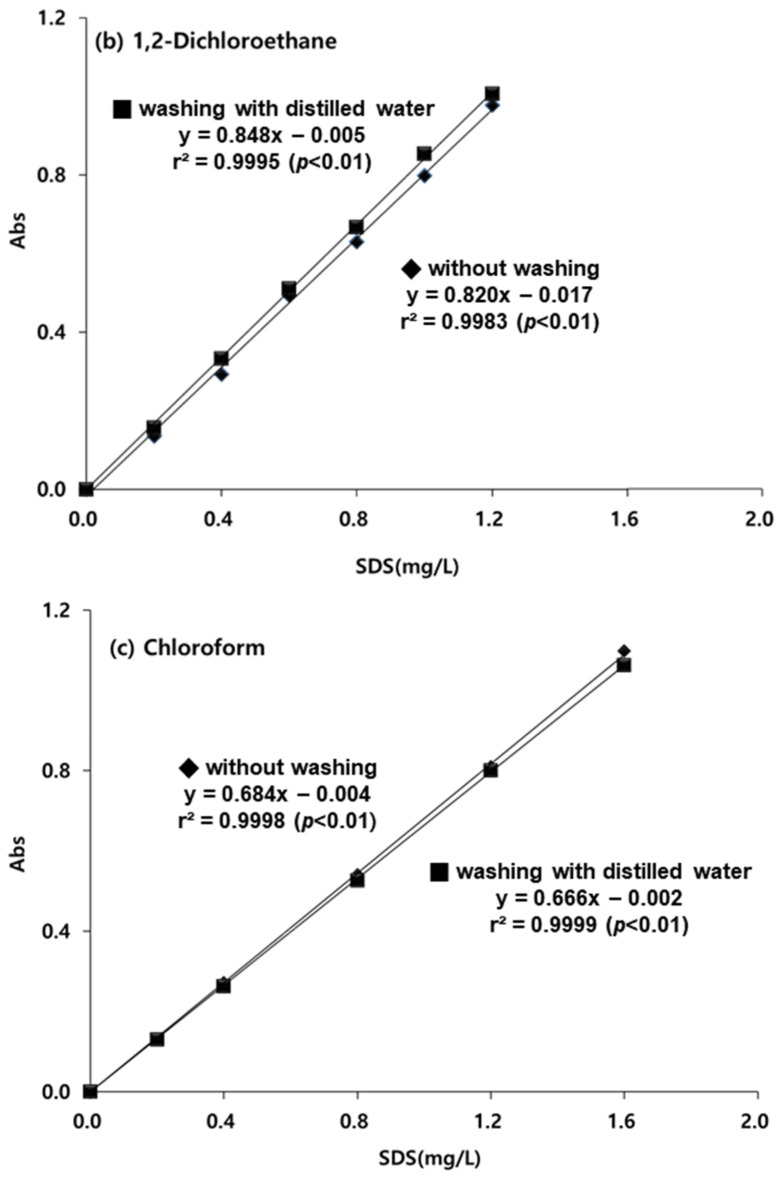
Comparison of washing and not washing on the extractability of sodium dodecyl sulfate (SDS) by (**a**) methyl isobutyl ketone (MIBK), (**b**) 1,2-dichloroethane (DCE), and (**c**) chloroform.

According to the official reference method using chloroform, the water-washing process is effective in reducing various interferences [29]. Thus, this study evaluated the effect of washing with distilled water on SDS extraction by MIBK and DCE, and the results were compared with those from chloroform (Figure 1). For the SDS standard curves, the slopes for washing and for not washing were not significantly different when MIBK, DCE, and chloroform were used. The slopes of standard curves for MIBK and DCE were compatible with the slope for chloroform. Consequently, the efficiencies of MIBK and DCE as organic solvents for SDS extraction were independent of the washing process. 

### 3.4. Development of MIBK-DCE Mixed Solvent

Results showed that both MIBK and DCE were the most suitable solvents for SDS extraction in water samples. However, the use of a single solvent, either MIBK or DCE, could not guarantee their superiority to chloroform (Table 1; Figure 1), and neither had physicochemical properties as an ideal extractant (Table 2). To achieve both a better extraction efficiency of the SDS and to have ideal conditions for solvents, we considered the mixed solvent of MIBK and DCE. 

To reduce the time for analysis, the mixing ratio of MIBK and DCE was assessed through preliminary tests to achieve a density for a mixed solvent of less than 1.0 g mL^−1^, because, during extraction and washing, the extraction solvent should have a lower density than that for water. As a result, at least 56.1% of the MIBK should be contained in the mixed organic solvents. Therefore, the mixed solvents were prepared using 60:40, 65:35, 70:30, 75:25, 80:20, 85:15, 90:10, 95:5, and 100:0 ratio of MIBK/DCE and analyzed according to the phase-separation time and SDS extraction efficiency.

As a result, the mixed organic solvents at 60:40 and 65:35 ratios of MIBK/DCE were not suitable, because the phases of water and mixed solvent were not clearly separated. A clear phase separation was observed at an MIBK concentration greater than 70% of the mixed solvents. Table 3 shows the SDS extraction efficiencies based on absorbance at different MIBK/DCE mixing ratios of ≥70:30, in comparison with those for chloroform. The mixed solvents, including 70:30, 75:25, and 80:20 ratios of MIBK/DCE, were effective as SDS extraction solvents. Additionally, when these mixed solvents (70:30~80:20 ratios of MIBK/DCE) were used, the phase-separation time was reduced from 15 min (time needed for chloroform) to 2.5~3 min. Therefore, when considering extraction efficiency of SDS, a 3:1 ratio of MIBK and DCE is recommended.

### 3.5. Effect of pH and Ionic Strength on the SDS Extraction Efficiency by MIBK-DCE (3:1) 

The efficiency of the extractant in the liquid–liquid extraction procedure is, in general, dependent upon the chemical properties of the extractant, which are mostly affected by pH and ionic strength. These factors affect the partitioning coefficient of analytes between the aqueous phase and organic phase, based on the rule of thumb ‘like dissolves like’ [3,10,43,44]. If a solvent in an AS analysis is independent of the pH of water samples, it can be applied to various environmental samples that may have a wide range of pH values. 

Table 4 shows the effect of the sample pH (2~11) on SDS extraction efficiency (recovery) by a MIBK-DCE (3:1) mixed solvent from a solution that was spiked with 1 mg L^−1^ SDS. There were no significant changes in the SDS extraction efficiency in pH ranges of 3~10. However, at pH 11, the extraction efficiency was slightly decreased, presumably due to the transformation of the methylene blue (MB) to dimethylthionoline, which resulted in the formation of pink colors in a chloroform phase [45]. Results indicated that a MIBK-DCE (3:1) mixed solvent can be applied for various water samples that have a wide range of pH values.

The official reference method for AS analysis [29,30] is known to have a limited application in seawater analysis (ionic strength of 0.7 M) due to interferences from high concentrations of Na and Cl [3,16,18,19,33,36,37,38]. Table 5 shows that the effects of ionic strength on SDS extraction efficiency by the MIBK-DCE (3:1) mixed solvent were insignificant, and SDS recoveries were above 96% even at an ionic strength of 2 M. It is speculated that, as the ionic strength is increased, the formation of MBAS is decreased due to decreased SDS activity. However, when the MIBK-DCE (3:1) solvent was used, results were independent of the ionic strength. Thus, it is expected that the MIBK-DCE (3:1) mixed solvent can be applied for analysis of anionic surfactants from a wide range of natural water samples including seawater samples. 

### 3.6. Interference of Cations on SDS Extraction Efficiency by MIBK-DCE (3:1) Mixed Solvent

When cations and anions co-exist with AS in water samples, they compete with AS for MBAS complex formation, resulting in a colorless complex between AS and MB [18,29,46]. Environmental water samples can contain various kinds of cations and anions at different concentrations. This can cause experimental errors, and, thus, it is necessary to study the interferences by cations and anions. 

Table 6 shows the effects of cations (Mg^2+^, NH_4_^+^, K^+^, Na^+^) on SDS extractability by the MIBK-DCE (3:1) mixed solvent, as compared to that in distilled water. The extraction efficiency was slightly decreased (2~3%) when cation concentrations were 0.1 M, and this concentration is hardly detectable in natural surface water. 

Magnitude of interference by Mg was slightly less than that of monovalent cations. This might be due to the fact that MB exists as MB+ when dissociated, which might show a higher competition with monovalent cations. There was no significant difference between K and Na. The results indicated that cations do not interfere in the MIBK-DCE (3:1) mixed solvent, and, thus, the solvent can be applied to extract AS from a wide range of natural water samples.

### 3.7. Interference of Anions on SDS Extraction Efficiency by MIBK-DCE (3:1) Mixed Solvent

Anions cause interference when the chloroform method is used, especially when the AS concentration is very low in water samples, because anions in natural water samples can form ion-pairs with the cationic MB(+) dye. This can result in a higher analytical value than the real concentration [3,16,18,19,29,33,37,46]. In this study, we used a wide range of monovalent anion sources to see their interferences on SDS extractability by MIBK-DCE (3:1) mixed solvent (Table 7). 

Among polyatomic ions, interferences were highest with NO_3_^−^, followed by NO_2_^−^ and CN^−^, but those by H_2_PO_4_^−^ and HCO_3_^−^ were relatively small compared to the former three anions. In case of carboxyl group anions, interferences by aromatic compounds were higher than those by aliphatic compounds. In aliphatic compounds, interferences seemed to be higher as numbers of COO^−^ groups increased. Among aromatic compounds, interferences were highest with salicylate, where both COOH and OH groups exist, followed by biphthalate, which has only one COOH group, and benzoate, which has no functional group. 

Considering the concentrations of anions commonly present in natural water samples and tested in this study, anionic interferences on SDS extractability by the MIBK-DCE (3:1) mixed solvent was considered to be minimal, except for the cases of halogen group ions such as I- and Br- that are presumably low in the natural water samples [47].

## 4. Conclusions

Criteria for an optimum solvent for the analysis of AS in aquatic samples include the following: a high AS extractability, a low volatility, a density lower than water, a quick separation time for the aqueous and solvent phases, and a low toxicity. The use of any single solvent screened in this study, including chloroform, could not meet these requirements. The combination of a MIBK-DCE (3:1) mixture satisfied such criteria and proved to be an ideal solvent for AS analysis. The extraction efficiency of SDS by MIBK-DCE (3:1) was superior to that of chloroform and was independent of pH, ionic strength, ionic interference, and washing process. The use of a MIBK-DCE (3:1) mixture could be applied for the determination of AS in various water samples, including seawater and industrial wastewater. The MIBK-DCE (3:1) mixture is a suitable alternative compared to traditional standard methods being used today. Development of a new analytical method for AS to replace chloroform, used in the conventional method, should be carried out.

## Figures and Tables

**Table 1:** Efficiency of six solvents for absorbance at the maximum absorption wavelength for SDS and phase-separation time.

Solvents	Phase-Separation Time	Maximum Absorbance Wavelength	Absorbance		
			Blank ^1^	Sample ^2^	BC ^3^
	min	nm			
Chloroform	12~22	652	0.0278	0.5377	0.5099
Dichloromethane	29~42	653	0.1507	0.8222	0.6715
1,2-Dichloroethane (DCE)	34~60	656	0.1145	0.7333	0.6188
n-Hexane	1~2	659	0.0025	0.0029	0.0004
Methyl isobutyl ketone (MIBK)	1~2	658	0.0278	0.5405	0.5127
Benzene	2~3	657	−0.0012	0.0363	0.0401

^1^ Distilled water without sodium dodecyl sulfate (SDS) at the maximum absorbance wavelength. ^2^ Spiked with SDS concentration at 0.8 mg/L at the maximum absorbance wavelength. ^3^ Blank compensated absorbance (BC) = (absorbance of the sample) − (absorbance of the control).

**Table 2:** Selected physicochemical properties of solvents used in this experiment.

Solvents	Density ^1^ (g/mL)	Health Hazard Group ^2^	Odor Threshold ^1^ (ppm)	Vapor Pressure ^1^ (mmHg, 20 °C)	Boiling Point ^1^ (°C)	Volatilization Rate ^3^ (mL min^−1^)
Chloroform	1.480	B2 ^4^	300	169	61	0.28
Dichloromethane	1.326	B2 ^4^	250	376	40	0.50
1,2-Dichloroethane	1.253	B2 ^4^	400	71	83.5	0.12
MIBK ^6^	0.801	D ^5^	8	16.5	116	0.04

^1^ Doumèche et al. [40]; Smallwood [41]. ^2^ U.S. EPA [42]. ^3^ Measured in this study. ^4^ Probable human carcinogen (sufficient evidence in animals). ^5^ Not classified as to human carcinogenicity. ^6^ Methyl isobutyl ketone.

**Table 3:** SDS extraction efficiency by MIBK-DCE at different mixing ratios and its comparison with chloroform.

Solvents	MIBK/DCE Mixing Ratios	Phase-Separation Time (min)	SDS Extraction Efficiency ^1^	
			Absorbances	CV ^2^ (%)
MIBK/DCE ^3^	100:0	1	0.3802 ± 0.0021	0.6
	95:5	1	0.4572 ± 0.0052	1.1
	90:10	1	0.5280 ± 0.0026	0.5
	85:15	1	0.5854 ± 0.0024	0.4
	80:20	2.5	0.6253 ± 0.0032	0.5
	75:25	2.5	0.6657 ± 0.0011	0.2
	70:30	3	0.6933 ± 0.0011	0.2
	0:100	87	0.8455 ± 0.0017	0.2
chloroform			0.6057 ± 0.0090	1.5

^1^ The spiked concentration of sodium dodecyl sulfate (SDS) was 1.0 mg L^−1^. ^2^ Coefficient of variation. ^3^ Methyl isobutyl ketone and 1,2-dichloroethane.

**Table 4:** Effect of pH on SDS extractability by MIBK-DCE mixture (3:1) solvent.

	pH									
	2	3	4	5	6	7	8	9	10	11
Concentration ^1^ (mg L^−1^)	1.04	1.01	1.01	1.02	1.02	1.01	1.03	1.02	1.02	0.94
SD ^2^	0.02	0.01	<0.10	0.01	<0.01	0.01	0.01	0.01	0.01	0.02
CV ^3^ (%)	2.1	1.4	0.4	0.7	0.3	0.9	0.4	0.6	1.0	2.2
Recovery (%)	103	100	100	101	102	100	102	101	101	93

^1^ The spiked concentration of sodium dodecyl sulfate (SDS) was 1.0 mg L^−1^. ^2^ Standard deviation. ^3^ Coefficient of variation (%).

**Table 5:** Effect of ionic strength on SDS extractability by MIBK-DCE mixture (3:1) solvent.

	Ionic Strength (M)								
	0	0.01	0.02	0.05	0.1	0.2	0.5	1.0	2.0
Concentration ^1^ (mg L^−1^)	1.00	0.99	0.99	0.99	0.99	0.98	0.98	0.97	0.96
SD ^2^	<0.01	<0.01	<0.01	0.01	<0.01	0.01	0.01	<0.01	0.01
Recovery (%)	100	99	99	99	99	98	97	97	96

^1^ The spiked concentration of sodium dodecyl sulfate (SDS) was 1.0 mg L^−1^. ^2^ Standard deviation.

**Table 6:** Cationic interference on extractability of SDS by MIBK-DCE mixture (3:1) solvent.

Compounds	Cations	Cation Conc. (M)	SDS Conc. ^1^ (mg L^−1^)	SD ^2^ (mg L^−1^)	Recovery (%)
Na_2_SO_4_	Na^+^	0.10	0.97 ^b,c^	0.01	97
K_2_SO_4_	K^+^	0.10	0.97 ^c^	0.01	97
(NH_4_)_2_SO_4_	NH_4_^+^	0.10	0.96 ^c^	<0.01	97
MgSO_4_	Mg^2+^	0.10	0.98 ^b^	<0.01	98
d-Water	-	~0	0.999 ^a^	<0.01	100

^1^ The spiked concentration of sodium dodecyl sulfate (SDS) was 1.0 mg L^−1^. ^2^ Standard deviation. The same letter on the column is not significantly different based on Tukey’s Studentized range test.

**Table 7:** Anionic interference on SDS extractability by MIBK-DCE mixture (3:1) solvent.

Compounds	Anions	Treated Anion Concentration (M)	Anionic Interference Conc. ^1^ (mg L^−1^)	Interference Strength (IS) ^2^
NaF	F^−^	0.5	0.049 ± 0.002 ^3^	3.4 × 10^−7^
NaCl	Cl^−^	0.5	0.628 ± 0.004	4.4 × 10^−6^
		1.0	0.972 ± 0.015	3.4 × 10^−6^
KBr	Br^−^	0.02	0.722 ± 0.012	1.3 × 10^−4^
		0.05	1.222 ± 0.053	8.6 × 10^−5^
KI	I^−^	0.0001	0.917 ± 0.010	3.2 × 10^−2^
		0.0002	1.250 ± 0.010	2.2 × 10^−2^
		0.0005	1.696 ± 0.017	1.2 × 10^−2^
NaNO_2_	NO_2_^−^	0.25	0.932 ± 0.011	1.3 × 10^−5^
		0.50	1.618 ± 0.016	1.1 × 10^−5^
KNO_3_	NO_3_^−^	0.0025	0.825 ± 0.007	1.2 × 10^−3^
		0.0050	1.300 ± 0.027	9.1 × 10^−4^
KCN	CN^−^	0.2	0.647 ± 0.046	1.1 × 10^−5^
		0.5	1.124 ± 0.137	7.9 × 10^−6^
KH_2_PO_4_	H_2_PO_4_^−^	1.0	0.174 ± 0.004	6.1 × 10^−7^
NaHCO_3_	HCO_3_^−^	1.0	0.143 ± 0.003	5.0 × 10^−7^
Sodium acetate	Acetate	1.0	0.203 ± 0.023	7.1 × 10^−7^
Sodium tartrate	Tartrate	0.5	0.162 ± 0.020	1.1 × 10^−6^
Trisodium citrate	Citrate	0.5	0.262 ± 0.036	1.8 × 10^−6^
Sodium benzoate	Benzoate	0.05	0.524 ± 0.018	3.7 × 10^−5^
Potassium biphthalate	Biphthalate	0.02	0.867 ± 0.031	1.5 × 10^−4^
		0.05	0.899 ± 0.123	6.5 × 10^−5^
Sodium salicylate	Salicylate	0.0002	1.082 ± 0.016	1.9 × 10^−2^
		0.0005	1.686 ± 0.027	1.2 × 10^−2^

^1^ Anionic interference concentrations were calculated from absorbance of the anionic solution by extrapolating them into the sodium dodecyl sulfate (SDS) standard curve. ^2^ Refer to Equation (1). ^3^ Standard deviation.

## Data Availability

Not applicable.
